# Preliminary development of an assay for detection of TERT expression, telomere length, and telomere elongation in single cells

**DOI:** 10.1371/journal.pone.0206525

**Published:** 2018-12-05

**Authors:** Ajay Ravindranathan, Beth Cimini, Morgan E. Diolaiti, Bradley A. Stohr

**Affiliations:** 1 Department of Pathology, University of California, San Francisco, California, United States of America; 2 Department of Biochemistry and Biophysics, University of California, San Francisco, California, United States of America; University of Nebraska Medical Center, UNITED STATES

## Abstract

The telomerase enzyme enables unlimited proliferation of most human cancer cells by elongating telomeres and preventing replicative senescence. Despite the critical importance of telomerase in cancer biology, challenges detecting telomerase activity and expression in individual cells have hindered the ability to study patterns of telomerase expression and function across heterogeneous cell populations. While sensitive assays to ascertain telomerase expression and function exist, these approaches have proven difficult to implement at the single cell level. Here, we validate in situ RNAscope detection of the telomerase TERT mRNA and couple this assay with our recently described TSQ1 method for in situ detection of telomere elongation. This approach enables detection of TERT expression, telomere length, and telomere elongation within individual cells of the population. Using this assay, we show that the heterogeneous telomere elongation observed across a HeLa cell population is in part driven by variable expression of the TERT gene. Furthermore, we show that the absence of detectable telomere elongation in some TERT-positive cells is the result of inhibition by the telomeric shelterin complex. This combined assay provides a new approach for understanding the integrated expression, function, and regulation of telomerase at the single cell level.

## Introduction

Human chromosomes are capped by telomeres, tandem arrays of TTAGGG repeats bound by a protective protein complex termed shelterin. The shelterin complex prevents telomeres from being recognized as DNA double strand breaks and from eliciting a DNA damage response. In addition, the shelterin complex regulates the recruitment of telomerase, an enzyme that maintains telomere length by adding new TTAGGG repeats [1].

As cells divide, telomeres shorten due to the inability of the DNA replication apparatus to fully replicate the ends of the chromosome [2]. Once telomeres are critically shortened, cell proliferation halts due to replicative senescence, apoptosis, or mitotic catastrophe, depending on the cellular context. Telomerase extends proliferative lifespan by maintaining telomere length, and it is estimated that 80–90% of all cancers depend on telomerase for their unlimited proliferative capacity [3].

The telomerase enzyme minimally consists of the protein reverse transcriptase component TERT and the template-containing RNA termed TERC [4]. TERC is diffusely expressed in cells, while TERT expression is more tightly regulated [5–7]. The correlation of TERT levels by RT-PCR [8] and that of telomerase activity by the Telomerase Rapid Amplification Protocol (TRAP) [9], together with the observation that ectopic TERT expression in telomerase negative cells is sufficient to confer telomerase activity [10–12], suggests that TERT protein is the primary rate-limiting component of telomerase activity in most bulk cell populations. However, it has been challenging to extend this work to the single cell level. While in situ detection of TERT mRNA has been reported in human tissue [13], the very low level of TERT expression in human cells makes it a challenging target for traditional in situ hybridization approaches [14]. Similarly, robust and reliable detection of TERT protein at the single cell level has been difficult due to the low expression levels of the protein. Finally, while telomerase activity can be easily assessed in bulk populations using the TRAP assay, the in situ version of this assay [15] has only been used sporadically due to difficulty implementing the technique. More recently, the development of a droplet digital PCR version of the TRAP assay (ddTRAP) has enabled sensitive single cell detection of telomerase activity. However, this assay cannot determine the amount of telomerase enzyme that traffics to and extends the telomeres in each cell, as it measures enzymatic activity based on elongation of an oligonucleotide telomeric primer substrate [16]. Altogether, there is relatively little information about telomerase function in single cells of heterogeneous populations.

We recently developed an in situ analysis method that uses incorporation of well-tolerated mutant telomeric repeats (Tolerated Sequence 1; TSQ1) to identify specific telomeres within a cell and specific cells within a population in which telomerase is adding new telomeric repeats [17]. Using the TSQ1 assay, we found that telomere elongation across a HeLa cell population is heterogeneous, with elongation detected in ~30% of the cells. In that study, we could not determine whether the heterogeneity was due to differential expression of telomerase or to the differential impact of positive and negative regulatory elements within individual cells of the population.

In order to determine the mechanistic basis of this heterogeneity, we describe here a new experimental approach that merges TSQ1 analysis with an in situ hybridization assay (RNAscope) capable of single molecule mRNA detection [18]. The combined assay enables detection of TERT expression, telomere length, and telomere elongation in individual cells. Using this assay, we demonstrate that the heterogeneous telomere elongation pattern observed in HeLa cells is in part due to variable expression of TERT mRNA. We further show that the absence of detectable telomere elongation in some TERT-positive cells is due to inhibition by the telomeric shelterin complex. Finally, we discuss the potential utility of this combined assay for further analysis of the function and regulation of telomerase at the single cell level.

## Materials and methods

### Cell culture

HeLa cervical carcinoma, Human LOX melanoma cells, MRC5 fetal lung fibroblasts and WI38 lung fibroblasts were grown in Dulbecco’s modified Eagle medium supplemented with 10% fetal bovine serum, 1% GlutaMax (Gibco) and 1% penicillin-streptomycin (Gibco). HCT116 colorectal cancer cells were grown in McCoy’s 5A media supplemented with 10% fetal bovine serum and 1% penicillin-streptomycin. Cells lines were obtained from ATCC (HeLa, HCT116), the UCSF Cell Culture Facility (MRC5, WI-38) and the laboratory of Elizabeth Blackburn (LOX). Cells were cultured at 37°C in a humidified incubator with 5% CO2. The HeLa TERT cells used in this study were generated by infecting HeLa cells with a retrovirus expressing TERT and a neomycin resistance gene. Infected cells were then selected with G418 for 7 days, sufficient to kill uninfected control cells treated in parallel [17].

### Retroviral POT1-ΔOB production and infection

Phoenix-AMPHO cells expressing amphotropic envelope protein (ATCC) were plated at a density of 6X10^6^ the day before transfection. Retrovirus particles were made by transfecting 12 mg of POT1-ΔOB (Addgene 13241) using Lipofectamine (Thermo-Fisher Scientific) and following manufacturer protocols. 48 hours after transfection, the media containing viral particles (10ml) was harvested and replaced with 10 ml fresh media. Following centrifugation to pellet loose cells, the supernatant was filtered through a 0.40μm filter and stored at 4°C. 72 hours later, the media was harvested, treated as before, pooled with the previous harvest, and stored in 5 ml aliquots at -80°C. The day before infection 1.7X10^5^ HeLa cells were seeded on a 10 cm plate and allowed to adhere overnight. The following day 4μg/ml of Polybrene, 4.5 ml of retrovirus and 0.5 ml of serum was added to the plates and incubated at 37°C. 8 hours later, an additional 5 ml of media was added and cells incubated for a further 48 hours. Following a media change, cells were selected with puromycin and propagated for a week before subsequent infection with TSQ1 or vector control (vec).

### Lentiviral TSQ1 production, infection and plating

Lentivirus for TSQ1 or vector control was produced as previously described using a second-generation lentiviral system [19, 20]. The day before infection 1.7X10^5^ HeLa, HeLa TERT or HeLa POT1-ΔOB cells were seeded on a 10 cm plate and allowed to adhere overnight. The following day 8μg/ml of Polybrene and 750 μL of virus was added to the plates. After 8 hours, the media was aspirated and replaced with fresh media. 200 μg/ml of hygromycin B was added 36 hours after infection for selection, which lasted until uninfected cells plated in parallel and treated with hygromycin B had died. Selected cells were then plated at a density of 3X10^4^ cells per well in 8 well chamber slides (Lab TEK II, Thermo-Fisher) previously coated with poly-lysine for 30 minutes and washed 3 times in PBS.

### RNAscope assay

8 well chamber slides containing plated cells were disassembled and processed according to manufacturer’s protocols using the RNAscope Brown kit and catalog probes for TERT and TERC (ACDBio). Following pre-treatment, probe hybridization and detection, the slides were stained in hematoxylin, dehydrated, cleared in xylene and mounted with Cytoseal XYL (Thermo Fisher).

### Combined RNAscope and peptide nucleic acid- fluorescent in situ hybridization (PNA-FISH) protocol

Slides were processed for RNAscope staining as per manufacturer protocols until the final dehydration step. Slides were then re-hydrated by washing in 70% and 50% ethanol for 5 min each. Following 2 5-min washes in phosphate buffered saline (PBS), the slides were fixed in 4% paraformaldehyde for 2 minutes, washed in PBS and permeabilized for 10 min in PBS+0.5% NP40. Slides were then rinsed in PBS and fixed with 4% paraformaldehyde for 2 more minutes. Following 2 washes in PBS, the slides were then treated with RNAse A (Qiagen cat #158922) diluted 1:100 in PBS for 2 hours. After 3 PBS washes the cells were treated with 0.1mg/ml pepsin in 0.1M HCl for 10 minutes at 37°C. Following 2 5-minute washes in PBS, the slides were fixed in 4% paraformaldehyde for 2 min, followed by 3 rinses in PBS. Cells were then dehydrated in an ethanol series (70%, 95% and 100%; 5 min each) and air-dried. Slides were then hybridized with PNA probes (Panagene) overnight at room temperature. The hybridization mixture contained 70% formamide, 10mM Tris pH 7.2, 1% blocking reagent (Roche catalog # 11096176001) and 0.5μg/ml of each PNA probe. The probes used were: Wild type telomere probe (FAM-OO-ccctaaccctaaccctaa), TSQ1 telomere probe (Cy3-OO-ccgcaaccgcaaccgcaa) and pan-centromere probe (Cy5-OO-cttcgttggaaacggga). Following hybridization, slides were washed twice in 70% formamide/ Tris HCl, pH 7.4 for 15 min each and three times in 0.05M Tris-HCl pH 7.4/0.15M NaCl/0.05% Tween 20 for 10 minutes each. Slides were dehydrated in an ethanol series as above, dried and cover-slipped with Prolong Gold with DAPI (Invitrogen catalog # P36931)

### Microscopy and image analysis

All images were obtained using the Metafer 4 slide-scanning platform (Metasystems) with a 63X 1.4 NA oil-immersion objective. Nuclei were first identified using a 10X objective and DAPI staining. The identified nuclei were subsequently imaged at 63X in both fluorescence (TSQ1, centromere, telomere) and brightfield (RNAscope) modes. RNAscope spots in each cell were counted manually. TSQ1 fluorescent foci (red channel)co-localizing with wild-type fluorescent telomeric foci (green channel) were counted manually. Based on comparisons with vector controls, we designated cells expressing 5 or more TSQ1 foci co-localizing with telomeric foci as TSQ1-positive. Counting and intensity analysis of telomeres were obtained using Cell Profiler software (www.cellprofiler.org). In brief, wild-type telomere length as reported in S5 Fig was determined by automated detection of the nucleus (based on DAPI signal) and the individual telomeric foci within each nucleus (green channel). The intensity of each telomere was measured, and the intensities of all the telomeres in a nucleus were used to calculate a mean telomeric intensity for that cell. Imaging pipelines are available upon request. Statistical and graphical analysis was performed using Excel (Microsoft) or Prism (GraphPad).

### Real time polymerase chain reaction

TERC expression levels were obtained as previously described [17, 21]: Superscript III (Thermo Fisher Scientific) and random hexamers were used to reverse transcribe TERC following on-column DNase digestion of the isolated RNA. QPCR was performed on the resulting cDNA using the Brilliant II qPCR kit (Agilent) using the following primers: TERC (FWD: ttgcggagggtgggcct; REV: cgggccagcagctgacatt) and Beta microglobulin (FWD:tcacgtcatccagcagagaatgga; REV: cacacggcaggcatactcatcttt). A one-step reaction was used to determine TERT levels using the Brilliant II qRT-PCR kit (Agilent) with TERT primers (FWD: cctgcactggctgatgagtgtg; REV: gatgctgcctgacctctgctt) and the control Beta microglobulin primers above, as previously described [17]. Reactions were run on a StepOnePlus real-time PCR system (Applied Biosystems). In each case data was normalized to control and expressed as a fold-change over baseline HeLa vec levels.

### Telomere restriction fragment analysis

Genomic DNA from HeLa cells and HeLa POT1-ΔOB cells was isolated using the Puregene kit (Qiagen) according to manufacturer protocols. Southern blotting of telomere restriction fragments was performed as described previously [22]. Briefly, genomic DNA was fragmented using Hinf1 and Rsa1 and DNA fragments separated on a 0.5% agarose gel. Following transfer of the DNA to HyBond membrane, the blot was probed with telomeric and ladder probes and exposed to X-ray film using chemiluminescent detection.

## Results

### The telomerase components TERT and TERC can be reliably detected using RNAscope

We chose to work with HeLa cells as a model system as they represent one of the few cell lines where telomerase dynamics have been previously assessed at the single cell level. Single cell cloning experiments suggested that significant heterogeneity exists both in terms of telomere length and telomerase activity within a HeLa population with stable mean overall telomere length [23]. Furthermore, a recent study using a TRAP assay variant based on droplet PCR suggested that ~30% of HeLa cells lack TRAP activity [16], and we have reported that the majority of telomere elongation in HeLa cells occurs within a small subset of cells [17]. Together, these results suggest that there is significant heterogeneity in both telomere length and telomerase activity within individual cells of a HeLa population, but the regulatory mechanisms underlying this heterogeneity are uncertain.

We hypothesized that the heterogeneous telomere elongation patterns seen in HeLa cells in our previous work would be driven at least in part by heterogeneous TERT expression. To assess TERT expression, we used RNAscope in situ hybridization detection [18, 24]. By employing extensive signal amplification, this proprietary technique enables detection of mRNA at the single molecule level.

**Fig 1:**
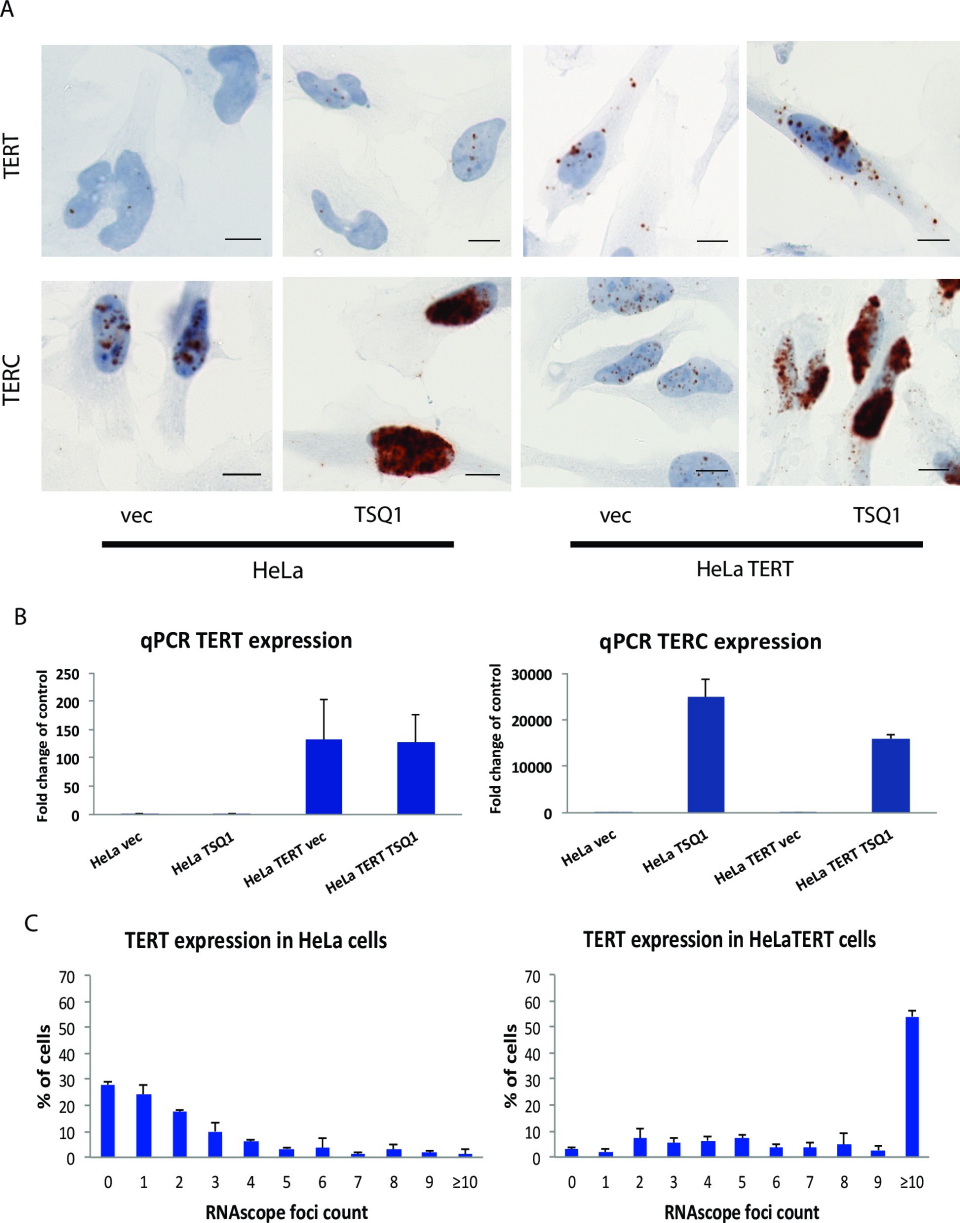
RNAscope detects TERC and TERT in heterogeneous cell populations. A. Representative images showing HeLa cells and HeLa cells overexpressing TERT (HeLa TERT), stained for TERT mRNA and TERC. Each of these cell types was infected with a lentiviral plasmid expressing the TSQ1 mutant TERC or a vector control (vec). Scale bars denote 10μM. B. qPCR to measure TERT and TERC expression in HeLa vec, HeLa TSQ1, HeLa TERT vec and HeLa TERT TSQ1 cells. In each case, levels are expressed as fold change over control HeLa vec levels. C. Frequency distribution of RNAscope TERT foci in populations of HeLa and HeLa TERT cells. The data is from 3 sets of biological replicates containing 100 cells each for either cell type.

**Fig 2:**
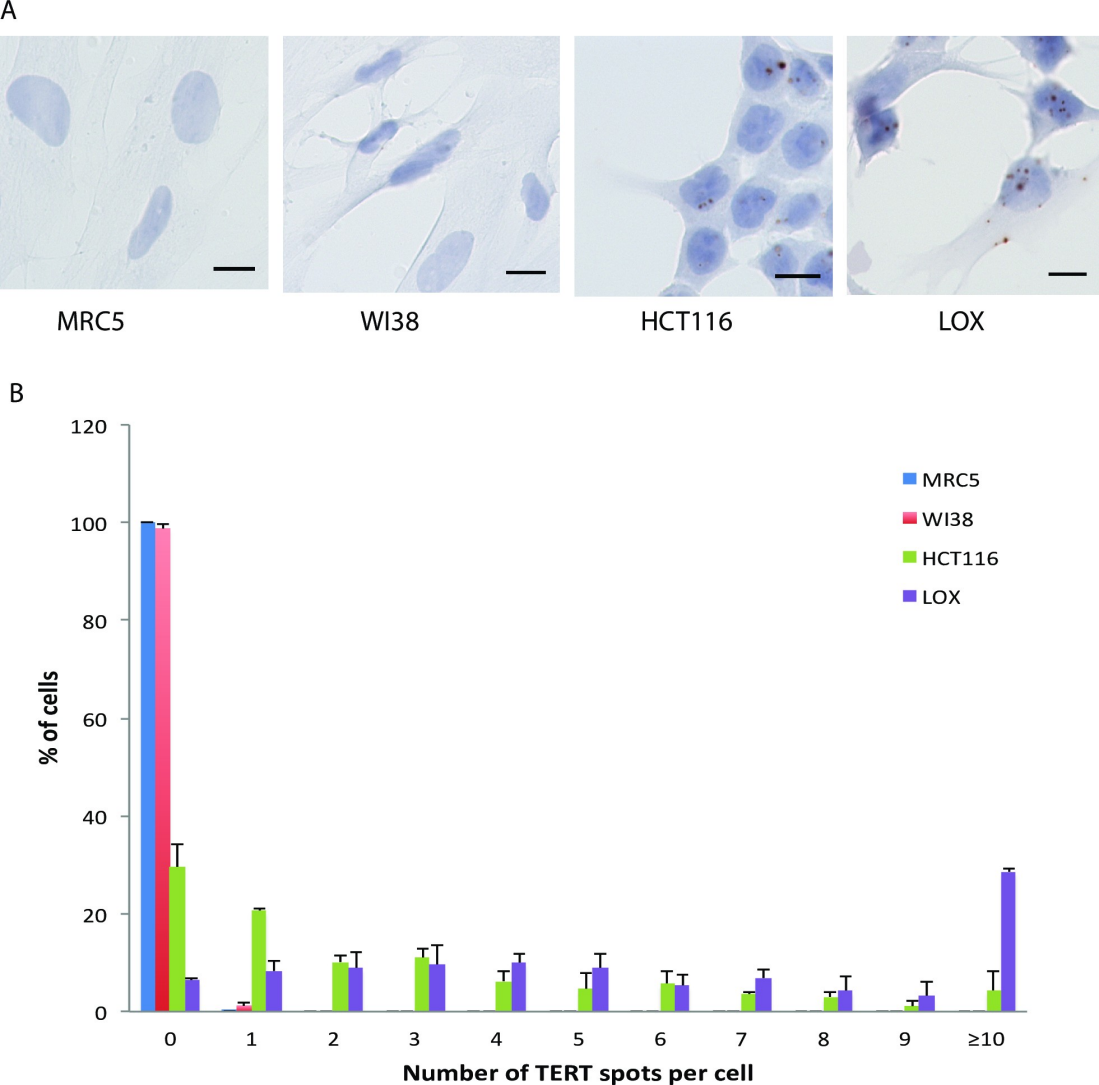
RNAscope controls. A. RNAscope for TERT expression in telomerase negative (MRC5 and WI38 fibroblast) and positive (HCT116 and LOX) cell lines. B. Frequency distribution of TERT RNAscope signals within populations of MRC5, WI38, HCT 116 and LOX cells. The data is from 3 sets of biological replicates each containing 100 cells for all cell types. Scale bars denote 10μM.

To validate RNAscope detection of telomerase components, we first tested RNAscope probes for TERC and TERT in control HeLa cells and in HeLa cells overexpressing TERT (HeLa TERT) and/or mutant TSQ1 TERC (HeLa TSQ1; HeLa TERT TSQ1). Consistent with published data demonstrating that TERC is ubiquitously expressed while TERT levels are typically rate limiting [5–7, 14], TERC expression was much higher than TERT expression in the control HeLa cell population (Fig 1). TERT expression was both low and heterogeneous, with approximately 30% of cells negative for TERT RNAscope. When either telomerase component was exogenously overexpressed, the RNAscope assay showed a corresponding increase in signal intensity. In each case, “vec” refers to the empty vector control for TSQ1 infection. The subcellular localization of the two telomerase components differed, with TERT mRNA detected in both nucleus and cytoplasm and TERC RNA predominantly seen in the nucleus. Taken together, these results demonstrate that RNAscope enables sensitive detection of both TERC and TERT in individual cells of a heterogeneous population. To further confirm the specificity of the RNAscope assay, we analyzed TERT expression in two cancer cell lines known to express telomerase (LOX and HCT116) [17, 25] and two fibroblast cell lines known to be telomerase negative (WI38 and MRC5) [26, 27]. Robust RNAscope TERT signal was observed in the cancer cell lines, while only extremely rare RNAscope TERT spots were seen in the fibroblast cell lines (Fig 2A and 2B).

To rule out that detection of genomic DNA, rather than mRNA transcripts, contributed to the nuclear TERT RNAscope signal, we ran the TERT RNAscope assay after RNAse-treating HeLa TERT cells post-fixation. RNAse treatment completely eliminated the TERT RNAscope signal, indicating that the RNAscope spots represent mRNA rather than genomic DNA (S1A Fig). Finally, since our ultimate goal was to combine TERT detection and PNA-FISH, we tested whether the brown RNAscope diaminobenzidine (DAB) reaction product was stable through the PNA-FISH protocol. We performed TERT RNAscope on HeLa TERT cells, imaged them in bright-field and subsequently performed PNA-FISH on the same slides. As shown in S1B Fig, the DAB reaction product is stable through the PNA-FISH procedure, although the hematoxylin staining intensity is reduced substantially post PNA-FISH.

### Combining RNAscope and PNA-FISH TSQ1 analysis enables detection of TERT expression, telomere length, and telomere elongation in single cells

**Fig 3:**
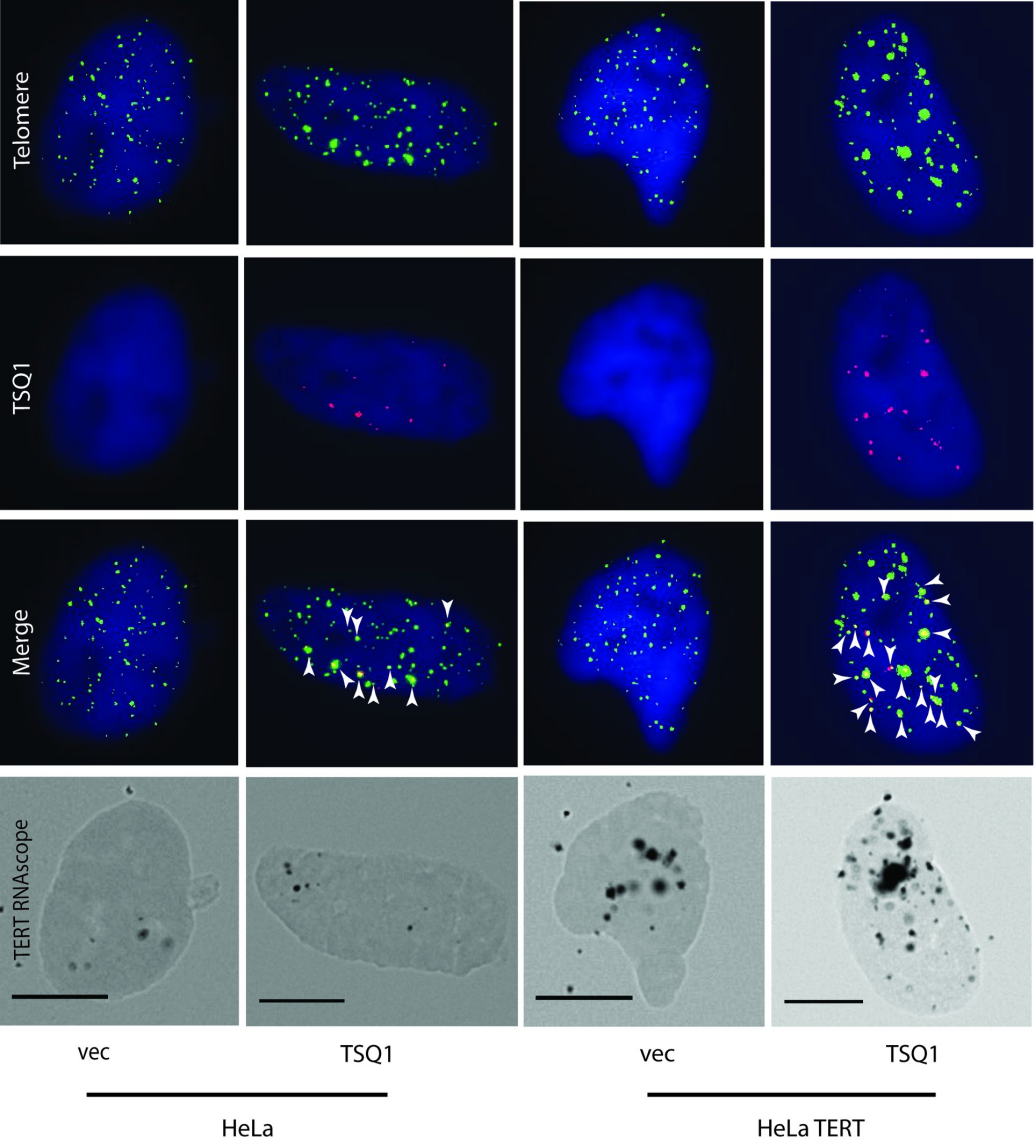
Combined RNAscope/PNA-FISH can be used to detect TERT expression and telomere elongation in single cells. Cells were assayed 7 days post-TSQ1 or vector infection. They were imaged via fluorescence for wild-type telomere (green) and TSQ1 (red) signal and then bright-field for the brown TERT DAB reaction product. TSQ1 spots co-localizing at telomeres are marked with arrows. Representative images are shown and scale bars denote 10μM.

**Fig 4:**
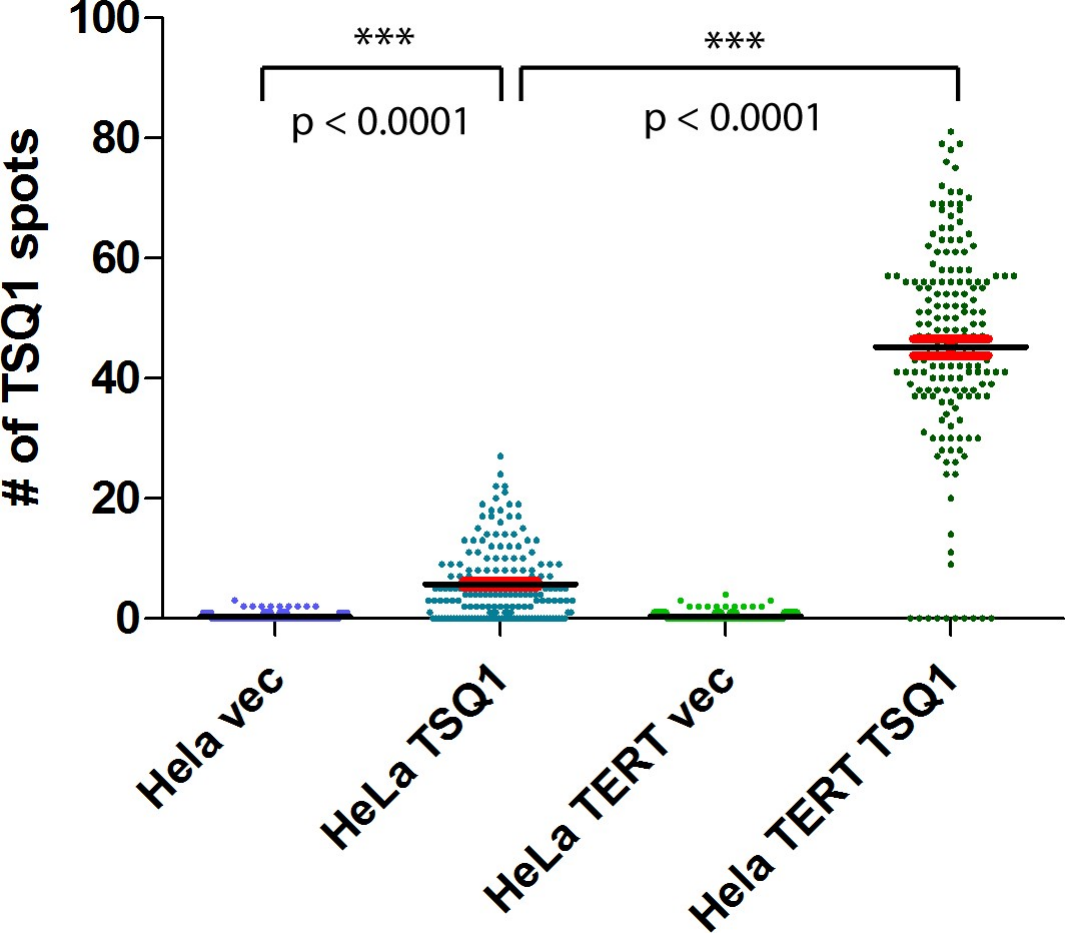
Quantification of TERT expression and telomere elongation in single cells. Swarm plots showing the number of TSQ1 spots co-localizing with telomeres in HeLa cells and HeLa cells overexpressing TERT. 171 cells from at least 2 separate experiments were assayed for each group. Means (horizontal black lines) and SEM (red lines) are shown for each population. P values (Mann-Whitney test) are provided and significant differences are marked with asterisks.

The observed heterogeneity of TERT RNA in HeLa cells parallels the heterogeneity of telomere elongation previously reported by our laboratory [17], suggesting that TERT levels may be an underlying driver of heterogeneous telomerase activity across the population. To test this hypothesis, we developed a combined RNAscope TSQ1 assay. In brief, TSQ1 TERC is overexpressed in HeLa or HeLa TERT cells for 5–7 days, after which RNAscope detection of TERT transcripts followed by fluorescence in situ hybridization detection of wild-type telomeric sequence (TTAGGG repeats) and TSQ1 telomeric sequence (TTGCGG) is performed. Subsequent brightfield and fluorescence imaging enables single cell detection of TERT mRNA (RNAscope spots in brightfield), telomere length (wild-type telomere fluorescence signal), and telomere elongation (TSQ1 telomere fluorescence signal). Representative images from the combined RNAscope/TSQ1 assay are shown in Fig 3, with additional images shown in S2 Fig. Incorporation of TSQ1 telomeric repeats, which reflects telomere elongation by telomerase, can be seen in both HeLa and HeLa TERT cells, but the degree of TSQ1 incorporation is much greater in the HeLa TERT cells (Fig 4). Altogether, 93% of HeLa TERT cells are positive for TSQ1 incorporation versus 44% of HeLa cells. Additionally, there are 4-fold more TSQ1 spots per TSQ1-positive cell in HeLa TERT cells versus HeLa cells (p<0.0001; Mann-Whitney test). In all, 171 cells from at least two independent experiments were assayed for each cell type to determine these data. Importantly, almost every cell treated with the TSQ1-expressing virus shows marked TERC overexpression relative to control (S3 Fig).

**Fig 5:**
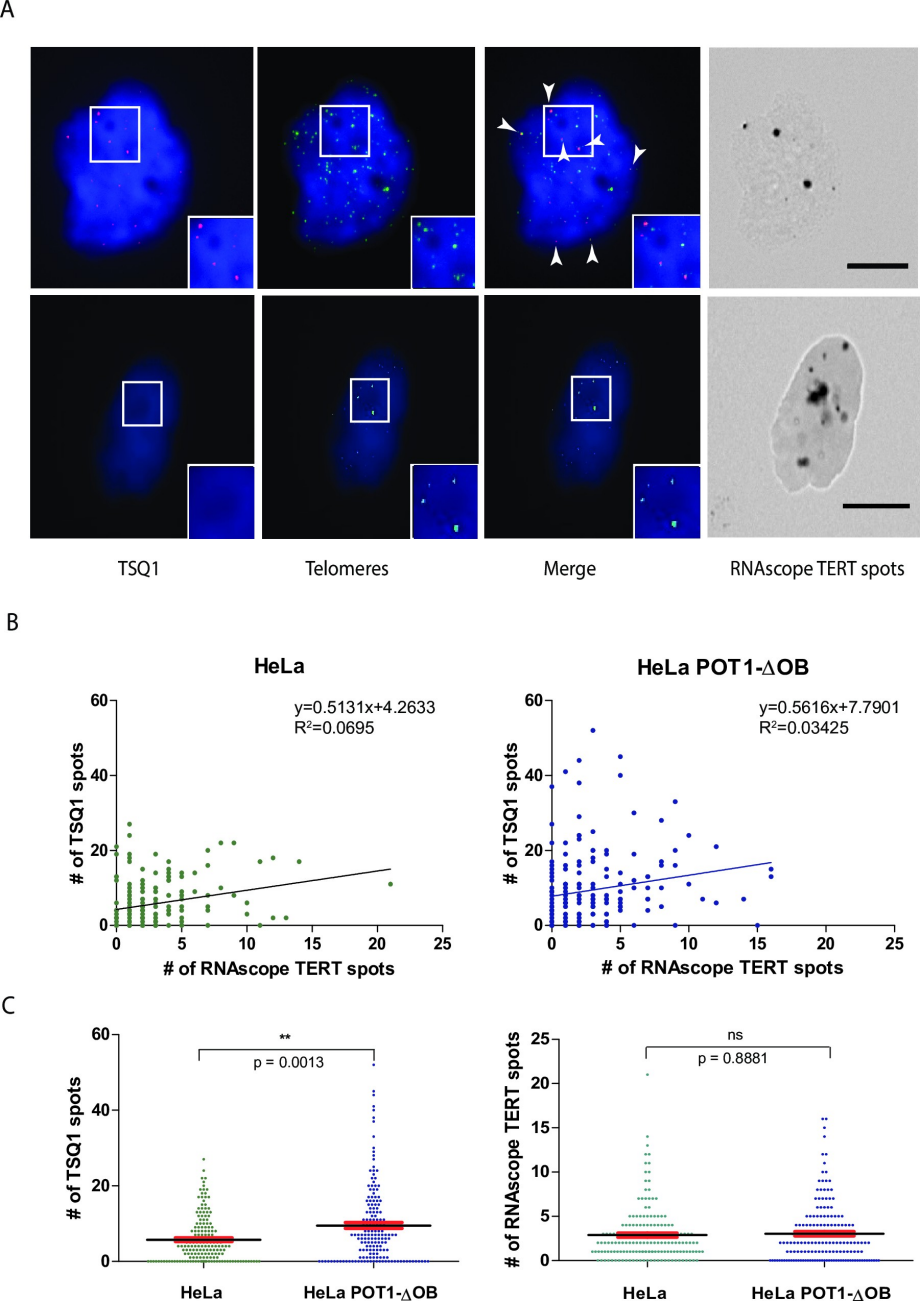
Correlation of TERT expression levels with incorporation of TSQ1 repeats in HeLa and HeLa POT1-ΔOB cells. A. Representative images of HeLa cells with robust TERT expression and either robust TSQ1 incorporation (upper panel of images) or no TSQ1 incorporation (lower panel of images). Arrows indicate TSQ1 spots that co-localize with telomeres. Enlarged images of the boxed regions are in the lower right-hand corner of each corresponding image. Scale bars denote 10μM. B Scatter plot demonstrating the correlation between TSQ1 incorporation and TERT expression in HeLa (left panel) and HeLa POT1-ΔOB cells (right panel). 171 cells from at least 2 separate experiments were assayed for each group. C. Swarm plots showing number of TSQ1 spots co-localizing with telomeres (left panel) and RNAscope TERT expression (right panel) in HeLa cells and HeLa cells overexpressing POT1-ΔOB. 171 cells from at least 2 separate experiments were assayed for each group. Means (horizontal black lines) and SEM (red lines) are shown for each population. P values are provided and significant differences are marked with asterisks, with ns denoting a non-significant difference. Of note, the HeLa cells quantified in this figure are the same as the cohort shown in Fig 4.

We further observed that some HeLa cells that express TERT show TSQ1 incorporation (Fig 5A, upper panels), while other cells show significant levels of RNAscope TERT signal but no detectable TSQ1 incorporation (Fig 5A, lower panels). Across the entire HeLa cell population, a weak but statistically significant correlation is seen between TSQ1 incorporation and RNAscope TERT levels (Spearman r = 0.275; p < 0.003) (Fig 5B, left panel), demonstrating that variable TERT expression is one determinant of the heterogeneous telomere elongation pattern observed in a HeLa cell population. However, a significant number of cells with TERT expression that is low or undetectable by RNAscope nevertheless shows robust TSQ1 incorporation at the telomeres, while many cells with multiple RNAscope TERT spots lack detectable TSQ1 incorporation. The presence of such cells strongly suggests that regulatory mechanisms other than TERT expression level also regulate the extent of telomerase function in individual cells of the population.

The absence of detectable telomere elongation in many of the TERT-positive cells may be due to negative regulation of telomerase activity. Several shelterin components have been implicated as negative regulators of telomere elongation [28], and overexpression of a mutant form of POT1 lacking the OB fold domain (POT1-ΔOB) disrupts this negative regulation and induces telomere elongation [29]. We tested the role of shelterin regulation by performing combined RNAscope/TSQ1 analysis in HeLa cells overexpressing POT1-ΔOB, in parallel with the HeLa cell experiment described in the paragraph above.

POT1-ΔOB overexpression induced bulk telomere lengthening as measured by Southern blot (S4 Fig), confirming its expected impact on telomere length regulation. Just like the control HeLa cells, the HeLa cells with POT1-ΔOB overexpression (Fig 5B., right panel) demonstrate a weak but statistically significant positive correlation between TERT expression as measured by RNAscope spot number and telomere elongation as measured by TSQ1 spot number. However, the average number of TSQ1 spots increased from 5.7 in HeLa cells to 9.4 in HeLa cells overexpressing POT1-ΔOB (p = 0.0013; Mann-Whitney test). This increase in TSQ1 incorporation was not the result of increased TERT expression, as the level of TERT RNAscope signal did not significantly differ (p = 0.8881; Mann-Whitney Test) between HeLa and HeLa POT1-ΔOB cells (Fig 5C, right panel). Altogether, this result suggests that the lack of robust TSQ1 incorporation in many TERT-positive cells is the result of negative regulation by the telomeric shelterin complex, and that disruption of this negative regulation leads to more telomerase-directed telomere elongation across many cells of the population.

Finally, we examined the correlation between the intensity of TERT RNAscope signal and telomere length in HeLa cells to determine if variable TERT RNA levels drive telomere length heterogeneity. We found no correlation between the number of RNAscope TERT spots in a cell and the average telomere length in that cell (S5A Fig). This observation is true with or without correction for centromere fluorescence intensity, indicating that differential probe access is not responsible for the observed result (S5B Fig). Thus, the telomere length in individual cells of the population does not correlate with TERT expression as measured at a single time point.

## Discussion

Despite a rapidly increasing understanding of human telomere biology, it has been difficult to monitor telomerase expression and function in individual cells of a population. Here, we validate and apply an assay that enables in situ detection of TERT expression, telomere length, and telomere elongation at the single cell level. Using the assay, we show that heterogeneous telomere elongation across a cell population is driven in part by variable TERT expression, and that telomere elongation in some TERT-positive cells is limited by negative regulation involving the telomeric shelterin complex. This combined assay will enable future investigations into the interplay of telomerase enzyme expression and function across cell populations.

By combining RNAscope detection of TERT transcripts with TSQ1 detection of telomere elongation, we can, for the first time, correlate expression of TERT with function of the enzyme at telomeres in individual cells of a population. Our previous work [17] showed that telomerase function is heterogeneous across a HeLa cell population, with only a subset of cells showing detectable TSQ1 incorporation. Here, we extend that work and show that variable TERT expression is partially responsible for the observed heterogeneity. Cells with detectable TERT expression are far more likely to incorporate TSQ1 than cells lacking RNAscope TERT signal [19].

While the presence of TERT expression shows a statistically significant positive correlation with TSQ1 incorporation, the strength of the correlation is weak, suggesting that factors other than TERT expression impact the extent of telomere elongation by telomerase. Many of the cells with TERT expression lack significant TSQ1 incorporation. We hypothesized that negative regulation of telomere elongation by the shelterin complex could explain this result, as a number of studies have shown that the 6-subunit shelterin complex plays an important role in telomere length control [1, 29–34]. Indeed, overexpression of POT1-ΔOB, which disrupts shelterin-mediated control of telomere length, resulted in significant TSQ1 incorporation in a higher percentage of TERT-positive cells. This result demonstrates that our single cell approach correlates well with prior bulk-culture experiments [29, 30] and provides a proof-of-principle demonstration that the assay can be used to interrogate regulators of telomere elongation at the single cell level.

Even with overexpression of POT1-ΔOB, the correlation between TERT expression and TSQ1 incorporation is weak, and there remain a number of TERT-positive cells that lack detectable TSQ1 incorporation. There are a number of possible explanations for this finding. First, it is likely that PNA FISH is not sensitive enough to detect TSQ1 incorporation below a certain threshold. While the vast majority of infected cells in our experiments highly overexpress TSQ1 TERC, a very small percentage of cells do not show high-level TSQ1 TERC overexpression despite a high viral titer and antibiotic selection, which could lead to reduced or absent TSQ1 signal in a very small subset of cells analyzed. A second possibility is that some cells are expressing inactive splice variants of TERT. Indeed, as in many human cancer cell lines, the majority of TERT mRNA in HeLa cells is reportedly the beta deletion variant, which codes for enzymatically inactive protein [35]. Currently, it is not possible to distinguish full-length TERT mRNA from inactive splice variants by RNAScope in our combined assay, so this hypothesis cannot be evaluated further. Finally, it is possible that the discrepancy between TERT expression and enzyme activity is due to other regulatory elements that limit activity of the enzyme. Unlike the TRAP assay, which measures telomerase elongation of a model substrate, the TSQ1 assay requires the telomerase enzyme to traffic to the telomere, access the telomere end, and elongate the native telomeric substrate. Numerous regulatory factors other than the shelterin complex modulate this pathway, and as a result, enzymatically active telomerase may be unable to access and elongate telomeres in some cells.

While the large majority of cells showing TSQ1 incorporation are also positive for TERT RNAscope, a small number of cells without detectable TERT mRNA show robust incorporation of TSQ1. One explanation for this counterintuitive result is that there is TERT mRNA present in these cells but that it is not detected by RNAscope hybridization due to degradation or some other technical issue. A second possibility is that TERT expression in individual cells of the population may be transient. Single cell cloning experiments with HeLa [23] found that some clonal populations converted from telomerase-negative to telomerase-positive, suggesting that TERT expression may fluctuate. In that case, TSQ1 incorporation could occur in TERT-positive cells, but the TERT mRNA may be gone by the time the RNAscope assay is performed.

While single cell assays are essential tools for understanding biological complexity, they pose a number of challenges in terms of experimental design and data interpretation, and the assay described here is no exception. Particularly challenging is the issue of false positive and negative results in individual cells, a problem that has been well documented in single cell genomic assays [36]. In our assay, we expect the false positive rate to be relatively limited, given the observed specificity of the RNAscope TERT probe. More problematic is the false negative rate, as we expect that low-level TSQ1 incorporation will be missed in some cells and at some telomeres due to the limits of FISH detection, and similarly, that RNAscope analysis will fail to detect low-level TERT expression in some cells due to factors such as mRNA degradation. It is difficult to accurately estimate false positive and negative rates, given that our assay has been expressly developed to link two biological variables–TERT expression and telomere elongation–that have been exceedingly difficult to reliably measure at the single cell level. Careful experimental design and interpretation can ameliorate the impact of this issue. First, as exemplified by our result with POT1-ΔOB overexpression, parallel analysis of cell populations can control for false positives and negatives and isolate the impact of a particular biological intervention. Second, analysis of a large number of individual cells can reduce the impact of false positives or negatives and enable identification of biologically meaningful patterns. For example, the correlation between TERT expression and telomere elongation that we identify would remain valid even in the presence of a significant false negative rate due to TERT mRNA or TSQ1 levels that are below the limit of detection. Thus, with careful experimental design and interpretation, the assay described here will enable interrogation of the mechanisms guiding telomerase function and telomere elongation at the single cell level.

Several other caveats of our approach bear brief mention. First, our technique relies on overexpression of TSQ1 TERC in the cells under study, which may itself impact the degree of telomerase activity in the cells. Although TERT is the limiting factor for telomerase activity in many situations, TERC overexpression may impact the degree of telomerase activity in some cases, and therefore the assay cannot be performed under true homeostatic conditions. Second, incorporation of variant TSQ1 telomeric repeats may impact subsequent telomere elongation by disrupting shelterin binding. In our prior work [17], we showed that incorporation of TSQ1 telomeric repeats is generally well-tolerated and does not induce the telomeric fusions typical of other variant telomeric repeat sequences. However, those results do not preclude other milder forms of telomeric disruption that might impact subsequent telomere elongation. In applying our assay, it is essential to consider these caveats carefully at the experimental design and data interpretation stages.

In this report, we have applied our assay in HeLa cells and demonstrated the role of particular factors–TERT expression and shelterin–in modulating telomere elongation patterns across a cell population. In the future, this assay could be applied to evaluate the role of other regulatory factors in guiding telomerase function. Telomerase biogenesis and trafficking is exceptionally complex, and there are numerous other cellular factors that may regulate the activity of telomerase in individual cells. Using our combined approach, factors regulating telomerase activity at the telomeres could be explored by analyzing the molecular differences between cells with strong TERT expression and strong TSQ1 incorporation versus cells with strong TERT expression and no detectable TSQ1 incorporation. Furthermore, this assay is applicable to a range of cultured cell types, and it will be of great interest to see how coordinated expression and function of telomerase vary in different cell types and under different growth conditions.

Finally, it has long been recognized that there is significant cell-to-cell variability in telomere length, but the mechanisms underlying this variability have not been identified. By taking advantage of RNAscope TERT detection, we have directly asked whether the level of TERT mRNA in each cell at a single time point correlates with the telomere length of that cell and have found no correlation between the two parameters. One possibility is that, as discussed above, TERT mRNA and protein expression fluctuate over time in individual cells of the population. In that case, the level of TERT mRNA in a cell at a single time point would not be expected to correlate with the telomere length of that cell. Alternatively, it is also possible that TERT expression remains relatively constant in each cell of the population, but that other regulatory factors modulate telomere elongation in the cell and determine the telomere length set-point of that cell. Finally, because telomeres shorten with each cell division, the proliferative history of each cell may influence its telomere length.

## Supporting information

S1 Fig RNAscope controls. A. TERT RNAscope analysis of HeLa TERT cells with or without RNAse A treatment prior to RNAscope analysis. B. TERT RNAscope reaction product before and after PNA FISH analysis in HeLa TERT cells, demonstrating the stability of the RNAscope reaction product. Scale bars denote 10μM.
(TIF)

S2 Fig TSQ1 co-localization in HeLa and HeLa TERT cells. HeLa or HeLa TERT cells were assayed 7 days post-TSQ1 or vector infection and imaged via fluorescence for wild-type telomere (green) and TSQ1 (red) signal. Arrows indicate TSQ1 spots that co-localize with telomeres. Representative images are shown and scale bars denote 10μM.
(TIF)

S3 Fig TERC expression in HeLa cells and HeLa cells over-expressing mutant TERC. RNAscope TERC staining on HeLa cells infected with lentiviral vector control or TSQ1.
(TIF)

S4 Fig Telomere elongation in HeLa POT1-ΔOB cells. Genomic blots of telomere restriction fragment length in HeLa and HeLa POT1-ΔOB cells 6 and 12 weeks after infection.
(TIF)

S5 Fig Correlation between TERT expression and telomere length in HeLa cells. Scattergram of TERT expression (number of RNAscope spots per cell) vs. mean telomere intensity values per cell, with and without correction for centromere intensity level. At least 150 HeLa cells were analyzed from at least 2 separate experiments.
(TIF)
