# Supplementary figures and images for: Preliminary development of an assay for detection of TERT expression, telomere length, and telomere elongation in single cells
Source: PLoS One. 2018 Dec 5;13(12):e0206525. doi: 10.1371/journal.pone.0206525 (PMC6281304; doi:10.1371/journal.pone.0206525)

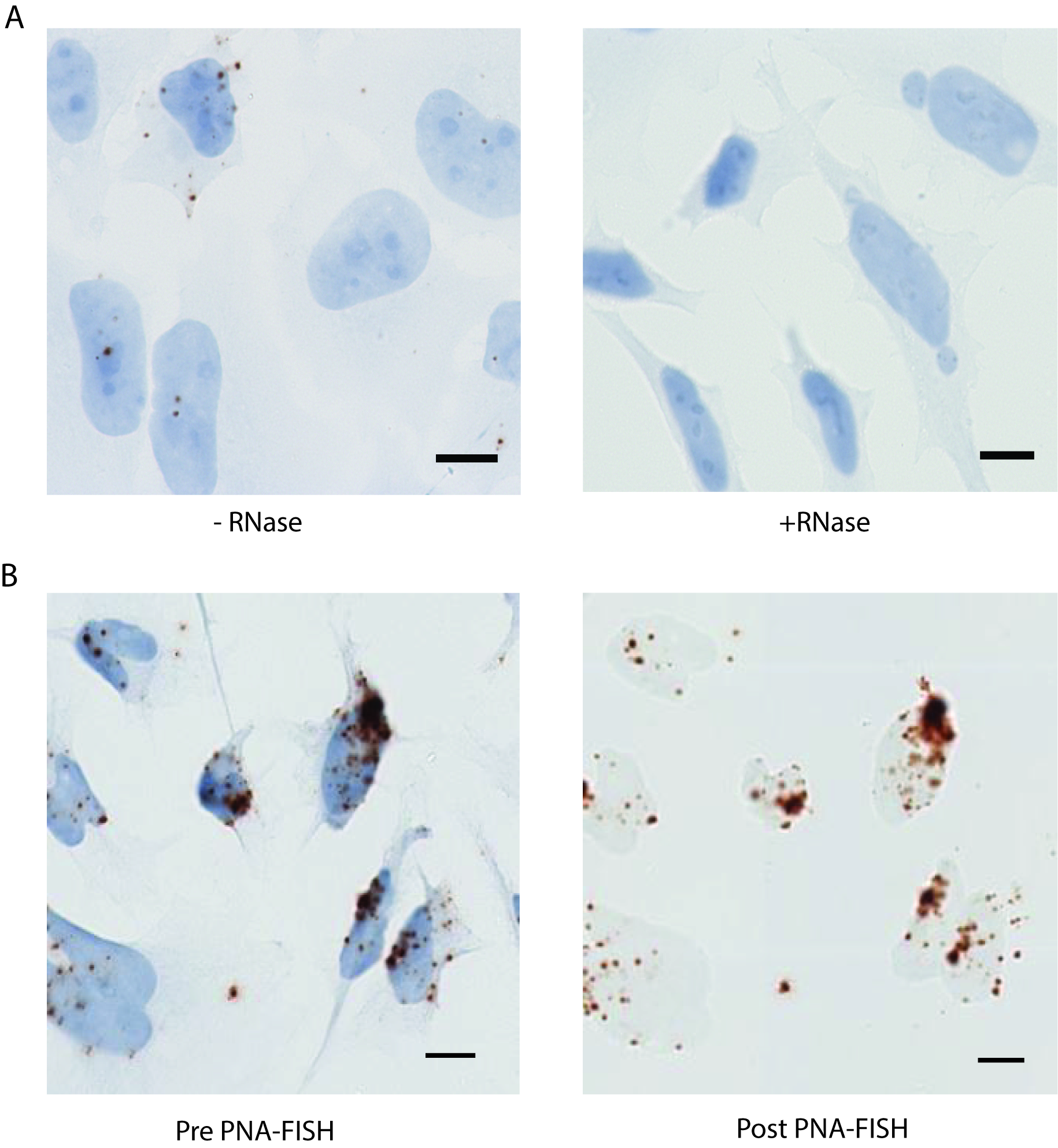

Supplement: S1 Fig — A. TERT RNAscope analysis of HeLa TERT cells with or without RNAse A treatment prior to RNAscope analysis. B. TERT RNAscope reaction product before and after PNA FISH analysis in HeLa TERT cells, demonstrating the stability of the RNAscope reaction product. Scale bars denote 10μM. (TIF) [file pone.0206525.s001.tif]

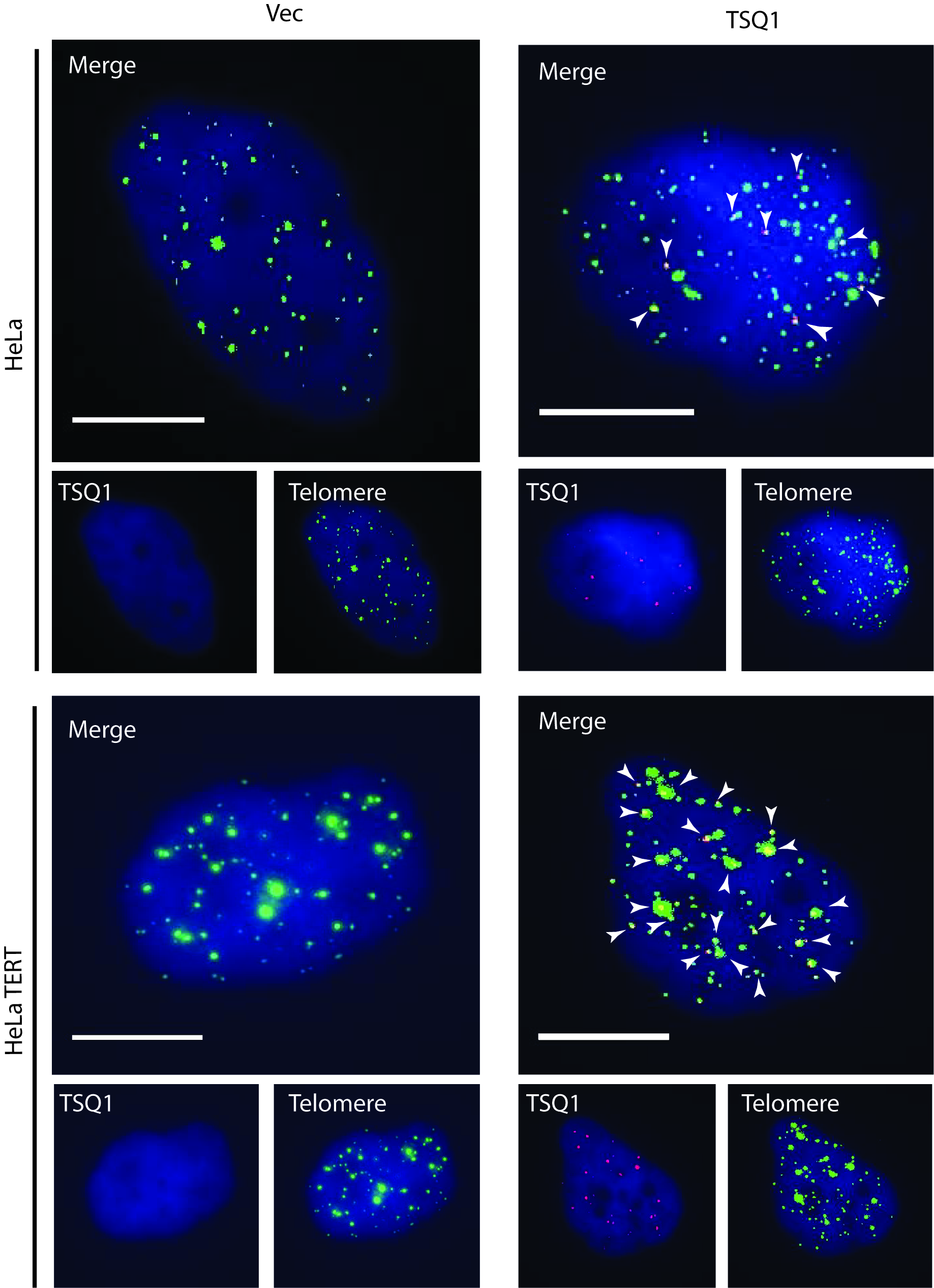

Supplement: S2 Fig — HeLa or HeLa TERT cells were assayed 7 days post-TSQ1 or vector infection and imaged via fluorescence for wild-type telomere (green) and TSQ1 (red) signal. Arrows indicate TSQ1 spots that co-localize with telomeres. Representative images are shown and scale bars denote 10μM. (TIF) [file pone.0206525.s002.tif]

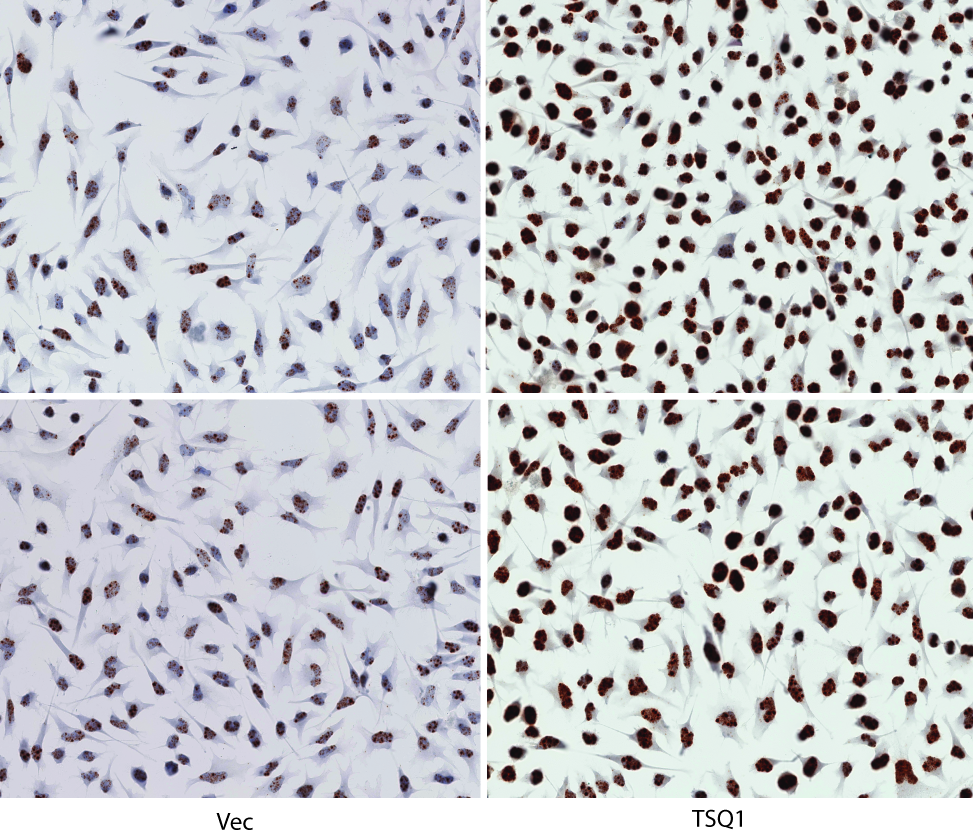

Supplement: S3 Fig — RNAscope TERC staining on HeLa cells infected with lentiviral vector control or TSQ1. (TIF) [file pone.0206525.s003.tif]

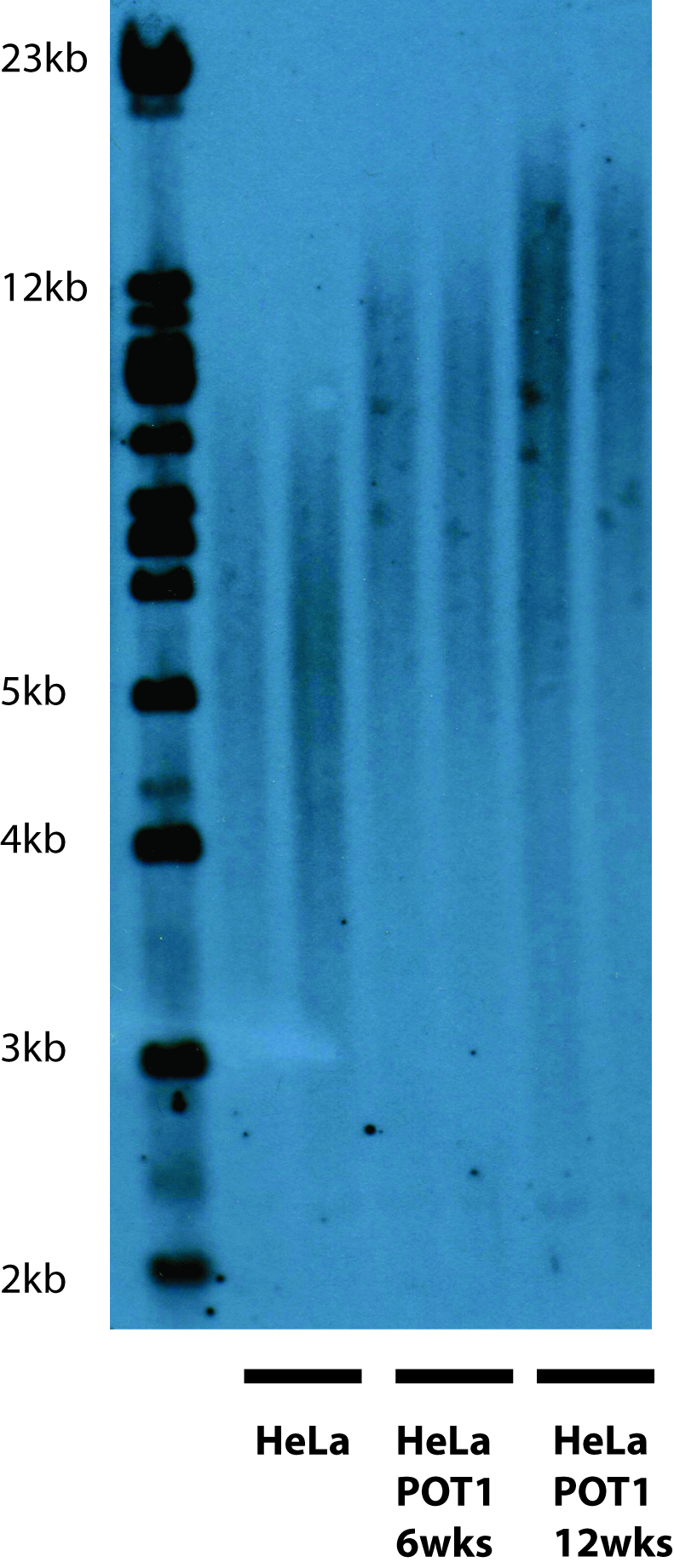

Supplement: S4 Fig — Genomic blots of telomere restriction fragment length in HeLa and HeLa POT1-ΔOB cells 6 and 12 weeks after infection. (TIF) [file pone.0206525.s004.tif]

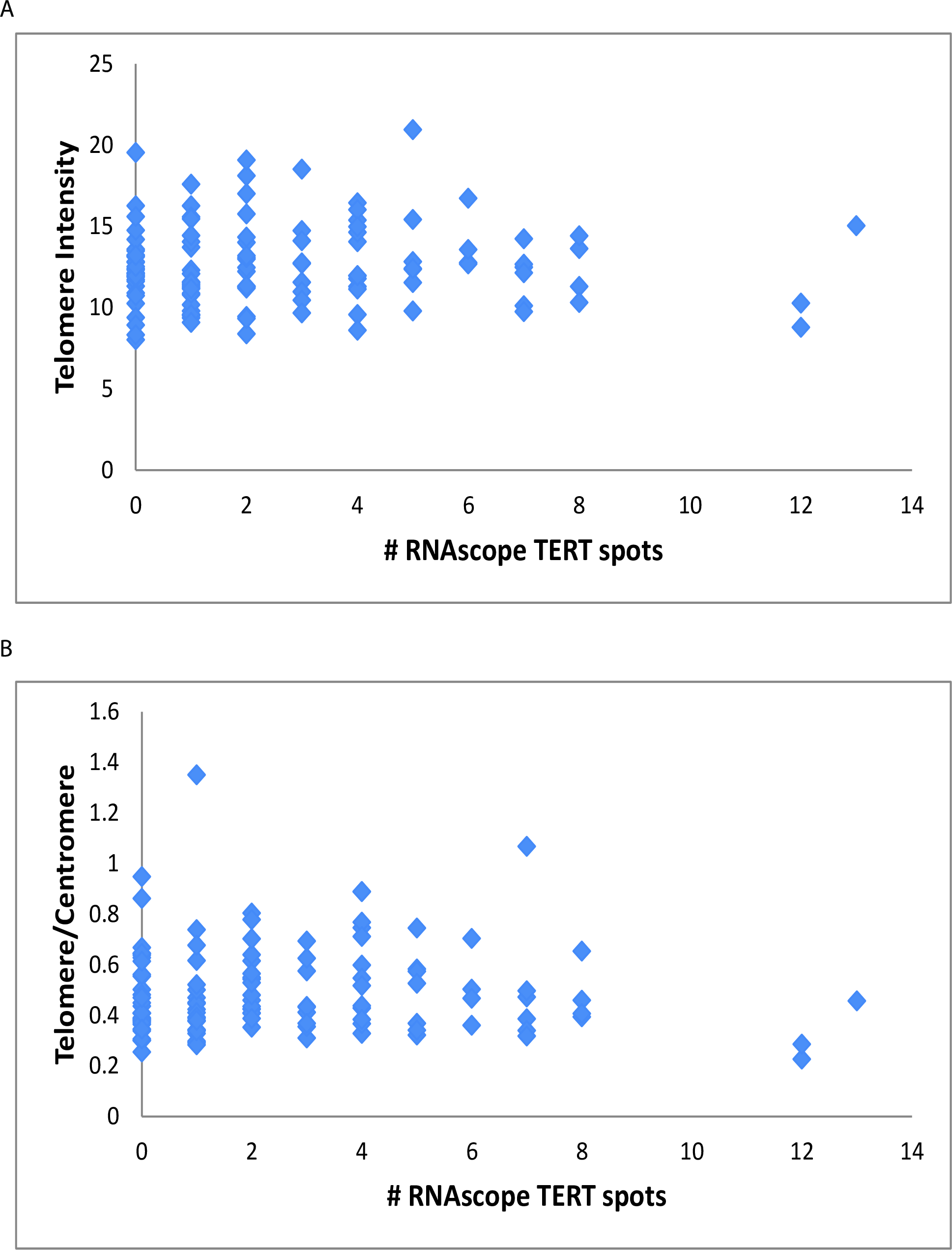

Supplement: S5 Fig — Scattergram of TERT expression (number of RNAscope spots per cell) vs. mean telomere intensity values per cell, with and without correction for centromere intensity level. At least 150 HeLa cells were analyzed from at least 2 separate experiments. (TIF) [file pone.0206525.s005.tif]
